# Seedless mutant ‘Wuzi Ougan’ (*Citrus suavissima* Hort. ex Tanaka ‘seedless’) and the wild type were compared by iTRAQ-based quantitative proteomics and integratedly analyzed with transcriptome to improve understanding of male sterility

**DOI:** 10.1186/s12863-018-0693-9

**Published:** 2018-11-20

**Authors:** Chi Zhang, Dihu Yu, Fuzhi Ke, Mimi Zhu, Jianguo Xu, Min Zhang

**Affiliations:** 1 State Key Laboratory of Subtropical Silviculture, Zhejiang A & F University, No.666, WuSu Street, Hangzhou, Zhejiang province People’s Republic of China 311300; 2 The Key Laboratory for Quality Improvement of Agricultural Products of Zhejiang Province, Zhejiang A & F University, Hangzhou, 311300 China; 3 Zhejiang Citrus Research Institute, Huangyan, 318020 China

**Keywords:** ‘Wuzi Ougan’, Male sterility, Proteome, Transcriptome, Phenylpropanoid metabolism

## Abstract

**Background:**

Bud mutation is a vital method of citrus. ‘Wuzi Ougan’ (mutant type, MT) as a bud variant of ‘Ougan’ (wild type, WT) was first found in 1996 and has become popular because of its male sterility and seedless character. Previous analysis of its cytological sections and transcriptome revealed that the abnormal microsporogenesis that occurs before the tetrad stage of anther development might be the result of down-regulated oxidation-reduction biological processes in MT. To reveal the mechanism behind the male sterility in MT at the post-transcriptional stage, proteome profiling and integrative analysis on previously obtained transcriptome and proteome data were performed in two strains.

**Results:**

The proteome profiling was performed by iTRAQ (isobaric Tags for relative and absolute quantitation) analysis and 6201 high-confidence proteins were identified, among which there were 487 differentially expressed proteins (DEPs) in one or more developmental stages of anthers between MT and WT. The main functional subcategories associated with the main category biological process into which the DEPs were classified were sporopollenin biosynthesis process and pollen exine formation. The enriched pathways were phenylpropanoid biosynthesis, flavonoid biosynthesis, and phenylalanine metabolism. Moreover, there were eight pathways linked in terms of being related to phenylpropanoid metabolism. Eighteen important genes related to phenylpropanoid metabolism were also analysized by qRT-PCR (quantitative real time PCR). An integrative analysis of the fold change at the transcript (log2 FPKM ratios) and protein (log1.2 iTRAQ ratios) levels was performed to reveal the consistency of gene expression at transcriptional and proteomic level. In general, the expression of genes and proteins tended to be positively correlated, in which the correlation coefficients were 0.3414 (all genes and all proteins) and 0.5686 (DEPs and according genes).

**Conclusion:**

This study is the first to offer a comprehensive understanding of the gene regulation in ‘Wuzi Ougan’ and its wild type, especially during the microsporocyte to meiosis stage. Specifically, the involved genes include those in phenylpropanoid biosynthesis, flavonoid biosynthesis, and phenylalanine metabolism, as determined by integrative transcriptome and proteome analysis.

**Electronic supplementary material:**

The online version of this article (10.1186/s12863-018-0693-9) contains supplementary material, which is available to authorized users.

## Background

China has an abundance of citrus resources and many cultivars derived from bud mutation [1]. Compared with other breeding methods, such as hybrid breeding, radiation-induced mutation breeding, and transgenic breeding, the advantages of bud mutation are the short breeding cycle and the fast breeding speed [2]. Therefore, genetic engineering has been applied to improve the quality of citrus cultivars by manipulating the molecular mechanism behind bud mutation. Mechanisms of bud mutation research have been focused on DNA methylation [3], retrotransposon insertion [4, 5], and on the structural and expressional difference of referred genes [6].

**Fig. 1:**
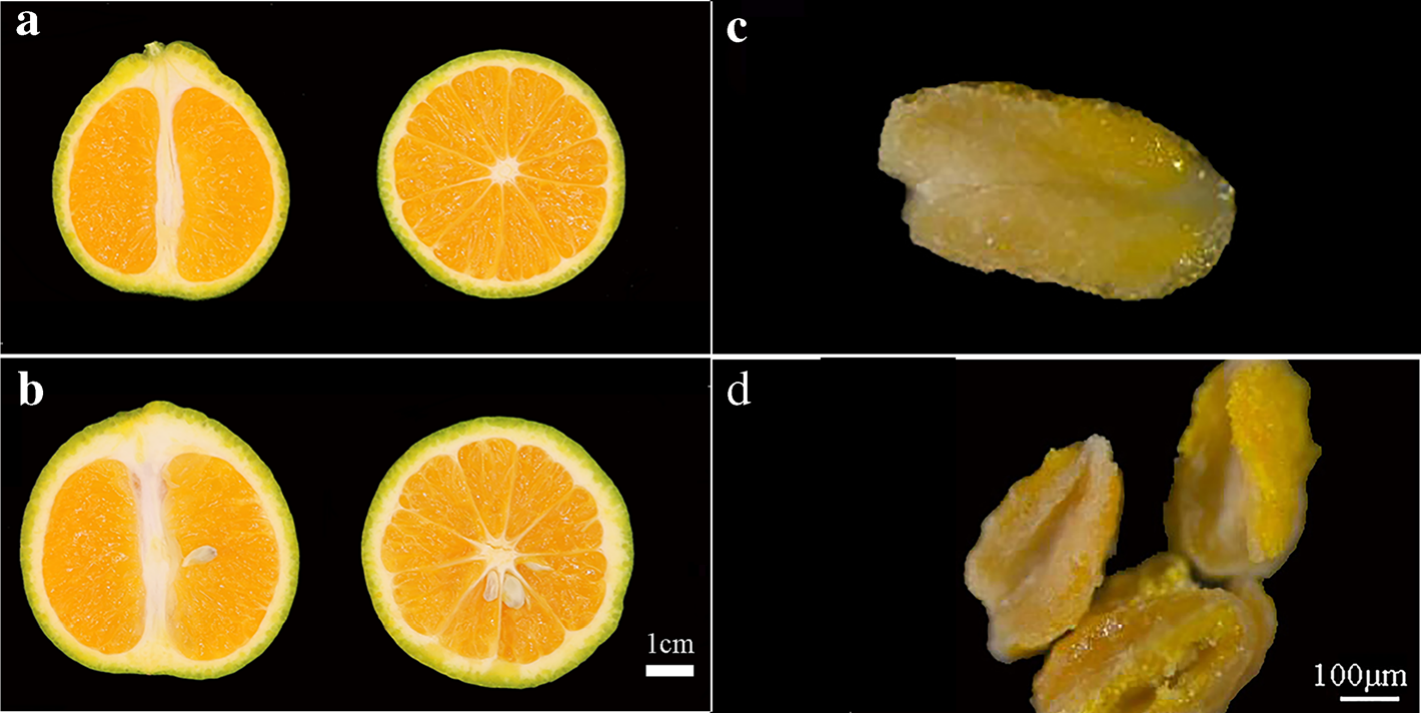
Morphological observation on cross section of fruit and mature anthers by stereoscope. The fruit was seedless in MT (**a**) and seedy in WT (**b**). The anther of MT (**c**) cannot be dehiscence spontaneously at mature stage when compared with WT (**d**)

‘Wuzi Ougan’ (*Citrus suavissima* ‘seedless’, mutant type, MT), a bud variant of ‘Ougan’ (*Citrus suavissima*, wild type, WT) (Fig. 1), has almost of the same excellent characteristics as WT except of seedlessness. Seedlessness is an important commercial feature for fresh and processed fruit in the citrus industry. Previously studies showed that male sterility was an important reason for seedlessness in ‘Wuzi Ougan’ [7, 8]. Failure staining on mature pollen by both KI-I_2_ and FDA suggested the pollen abortion [7]. Further observation by transmission electron microscopy (TEM) found that empty pollen grains were revealed during pollen maturation [7, 8]. In addition, microspore mother cells were found to be abnormal at the tetrad stage by scanning electron microscopy (SEM) [8] and suggested that the problem led to male sterility might occur at meiosis or earlier. Follow the observations, *CsRad51*, which was the gene in charge of double strand break (DSB) formation and DNA damage repair, was detected overexpression by qRT-PCR during microsporocytes to meiosis stage of anthers in MT when compared with WT [9]. Lacking of DSBs was reported to be involved in failure to synapsis [10, 11]. However, both excess of DSBs and *AtRad51* expression could be induced by radiation [12].

Male Sterility induced by genes (nuclear and cytoplasmic) mutation is genetic and widely documented in higher plants [13]. In general, male sterility is characterized by failure of pollen grain development or function, and is either controlled by nuclear genes alone (genic male sterility) or regulated by the complementary action of nuclear and cytoplasmic genes (genetic-cytoplasmic male sterility) [13]. In citrus, male sterility occurred naturally in bud sports [7, 8, 14, 15] or synthesized artificially by somatic protoplast fusion [16–22]. Those mutations and hybrids/cybrids exhibited usually failures of stamen development [23, 24] and some were evaluated by focusing on anther development [7, 8, 25, 26]. In addition to those with genic male sterility, some citrus cultivars with genetic-cytoplasmic male sterility exhibited the incidence of aborted anther of hybrid seedlings fits the segregation criterion controlled by major genes [25, 27]. Therefore, both male sterile types, those are conditioned by genes from either nuclear or nuclear organelle (eg. mutations of mitochondrial genes), could be characterized by genetically expression on RNA and protein level. In addition to sophisticated cell fusion, cross hybridization is attractively alternative and facilitated approach on introducing male sterility in seedless breeding of citrus.

In this connection, exploring efforts for improve understanding of post-transcriptional and -translational profiles have increased in citrus. Recently, vast of metabolic pathways and biological processes, as well as included genes and proteins, were reported in male sterility cultivars or lines [22, 28–33]. Among those mentioned in reports, genes or proteins have been focusing on the phenylpropanoid metabolism pathways. Phenylpropanoids metabolism is comprehensive network and includes several pathways and key enzymes. PAL (Phenylalanine ammonia-lyase) catalyzes the first step in the biosynthesis of the phenylpropanoid skeleton. PAL is documented to express predominantly in anthers [34–36] and induce sterile pollen when reduce the activity [37]. Flavonoids biosynthesis is leading pathway located in phenylpropanoids metabolism. CHS (chalcone synthase) and CHI (chalcone isomerase) are rate-limiting enzymes and involved in male sterility when their expression were down-regulated [38–40]. Lignin biosynthesis is the last steps in phenylpropanoids network. CCR and CAD are essential for the monolignol pathway and confirmed by triple mutant of *ccc* exhibiting male sterility and severe dwarf phenotype [41].

Integrative analysis has become an important method to deeply explore the origin of the phenomenon. It has been applied to studying the mechanisms of fruit ripening [42], analyzing the regulatory roles of transcription factors [43], and comparing different breeds [44]. In the present study, proteome profiling and integrative analysis combined with the use of previous transcriptome data obtained by RNA-Seq were performed to identify the candidate pathways and genes related to male sterility in ‘Wuzi Ougan’. A focus was placed on a pathway that potentially plays a leading role in male sterility in ‘Wuzi Ougan’, namely, phenylpropanoid metabolism. The obtained results should improve our understanding of male sterility in citrus.

## Methods

### Sample preparation and data source

‘Wuzi Ougan’ **(***Citrus suavissima* Hort. ex Tanaka ‘seedless’**)** and its wild type were planted at Jin Chao Gang farm, Wenzhou, Zhejiang, China. The materials used for iTRAQ analysis were collected from three stages (S1, S2, S3) according to the length of floral bud [9]: Stage 1 (the flower bud of the sporogonium, 1.2 mm < floral bud length < 1.6 mm), Stage 2 (the anther of the early microsporocyte, 2.0 mm < floral bud length < 2.4 mm) and Stage 3 (the anther of microsporocyte to meiosis, 2.4 mm < floral bud length < 2.8 mm). The flower buds were too small to form anthers and we collected the flower bud in Stage 1. Three independent technical replicates were set in each stage. The materials of qRT-PCR were collected from four stages according to the length of floral bud: I (the early microsporocyte, 2.0 mm < floral bud length < 2.4 mm), II (the microsporocyte to meiosis, 2.4 mm < floral bud length < 2.8 mm), III (the Tetrad, 2.8 mm < floral bud length < 3.1 mm) and IV (the pollen maturation, 6.5 mm < floral bud length < 7.1 mm). Anthers at microsporocytes were collected from ‘Wuzi Ougan’ and its wild type respectively for RNA-Seq. Raw sequencing reads obtained by transcriptomic sequencing were deposited in the Sequence Read Archive (SRA) database in NCBI under the accession number of PRJNA430695 (https://www.ncbi.nlm.nih.gov/bioproject/?term=PRJNA430695).

### Protein extraction, iTRAQ labeled and LC-MS/MS

TCA-acetone method as an important method was selected to extract protein [45]. The method of FASP Digestion according to universal sample preparation method for proteome analysis [46]. iTRAQ labeled peptides were fractionated by SCX chromatography using the AKTA Purifier system (GE Healthcare) [47–50]. LC-MS/MS analysis was performed on a Q Exactive mass spectrometer (Thermo Scientific) that was coupled to Easy nLC (Proxeon Biosystems, now Thermo Fisher Scientific) for 60 min. The mass spectrometer was operated in positive ion mode. MS/MS spectra were searched using MASCOT engine (Matrix Science, London, UK; version 2.2) embedded into Proteome Discoverer 1.4 and the parameters were set in Table. 1.

**Table 1:** The parameters Proteome of Discoverer 1.4

Item	value
Enzyme	Trypsin
Max Missed Cleavages	2
Fixed modifications	Carbamidomethyl (C), iTRAQ4/8plex (N-term), iTRAQ 4/8plex (K)
Variable modifications	Oxidation (M), iTRAQ 4/8plex (Y)
Peptide Mass Tolerance	± 20 ppm
Fragment Mass Tolerance	0.1 Da
Database pattern	Decoy
Peptide FDR	≤0.01
Protein Quantification	The protein ratios are calculated as the median of only unique peptides of the protein
Experimental Bias	Normalizes all peptide ratios by the median protein ratio. The median protein ratio should be 1 after the normalization.

### Data analysis

The protein sequences of differentially expressed proteins were in batches retrieved from UniProtKB database in FASTA format. The retrieved sequences were locally searched against SwissProt database Citrus clementina (https://phytozome.jgi.doe.gov/pz/portal.html#!info?alias=Org_Cclementina) to search homologue sequences from which the functional annotation can be transferred to the studied sequences. The final iTRAQ ratios of proteins were then normalized by the median average protein ratio of the equal mix of different labeled samples [28]. In this work, each sequence was retrieved and loaded into Blast2GO (Version 3.3.5) for GO mapping and annotation. The FASTA protein sequences of differentially changed proteins were blasted against the online Kyoto Encyclopedia of Genes and Genomes (KEGG) database (http://geneontology.org/) to retrieve their KOs and were subsequently mapped to pathways in KEGG.

### Quantitative real-time PCR (qRT-PCR) analysis

Eighteen DEPs selected from iTRAQ results were analyzed by using qRT-PCR. Total RNA was extracted from anther of ‘Ougan’ and ‘Wuzi Ougan’ in four stages (I, II, III, IV). The primers were designed by the GenScript Real-time PCR (TaqMan) Primer Design Center (https://www.genscript.com) (Table. 2). The Actin gene *GU911361* as a reference was used to calculate the relative fold-differences based on comparative cycle threshold (2^-ΔΔCt^) values [51].

**Table 2:** The primer sequences for Real-time PCR

Gene ID	Primer sequence (forward)	Primer sequence(reverse)
Ciclev10011520m	CTAAGGTTGCACCCACCAAC	GGCCTTTCATGTCCACATCC
Ciclev10027912m	TGTTCCGAGCTCCAGTTTCT	TCTCCTCCAAGTGCCTCAAG
Ciclev10011175m	TACTCCGGCATCCGATTTGA	CCGCTTGTTTAGAGGCTTCC
Ciclev10025933m	CTATGGTTGCCGATGAGCAC	GCCTTTGCGAATTTGACAGC
Ciclev10028718m	GATGGCAAGCCTACACAAGG	CCGCTCTGCTTCAATCCAAA
Ciclev10001726m	GGCAACTGCTTCAGCTTCTT	ACCTGAACGAAGCAATCGTG
Ciclev10015790m	AAGGCCTTCTGCAGAGTGAT	TGACCCTCCTGCAGTTCAAT
Ciclev10032081m	CGACTGCTTCGTTGAGGTTT	TCTGAATCCCTTTCGGCCTT
Ciclev10015870m	TGGTGTTGTTTCCTGTGCTG	TAAGGGCCTCCAAGCTGAAA
Ciclev10020821m	TCACATGCAATGGGACTTGC	TGGCTGCAGCTTCAGGATTA
Ciclev10031951m	TGATTCATTGTCCCGGTGGA	TGCTCGTCTTTGGGAGACAT
Ciclev10029158m	CGACAACACCCTATGGAACG	GACGGCAGATAGTGACTCCA
Ciclev10031133m	TCGTGATGAACCCAAATGCC	GGCTGCGTCAGATTTCACTT
Ciclev10013148m	TCCACTGTCGCCAACATCAT	TTGGGTACCGACAGCTTCTT
Ciclev10015535m	GAAGCTCGGGAAAGAAGCTG	AGTCTTTAGCGAGGCGAAGA
Ciclev10032697m	GGGAAGCCACTTAGCCACTA	GAGCTAAGGCGACGATGTTC
Ciclev10031286m	CTGAAGCCGAAGCTACCAAC	TTCCCGTGCTCTCTTCCTTT
Ciclev10025088m	ATTAAGGAGCCAACGGCAAC	TACACACCTTCCACCTGACC
GU911361(*Citrus sinensis*)	ATCTGCTGGAAGGTGCTGAG	CCAAGCAGCATGAAGATCAA

## Results

### The iTRAQ analysis

**Fig. 2:**
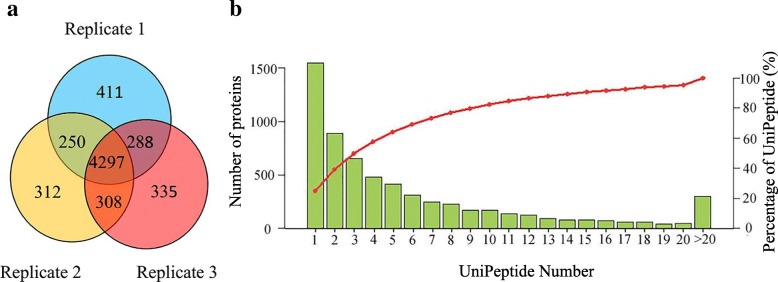
Identification and analysis of anther proteome in ‘Ougan’ and ‘Wuzi Ougan’. **a** Number of proteins identified from the triple iTRAQ proteomic experiments. **b** Number of proteins that match corresponding unique peptides (unipeptide) number

Proteins play a role in most cellular functions and processes, and proteomics offers a direct and integrated perspective on cellular processes and networks. Thus, proteomic analysis of the anthers of MT and WT was performed using iTRAQ to explain CMS. Three developmental stages (the sporogonium, the early microsporocyte, the microsporocyte to meiosis) were analyzed in technical replicates. More than 380,000 mass spectra were collected from each replicate in this study. By performing data filtering to eliminate low-scoring spectra, 25,108, 24,243, and 24,238 unique peptides were collected from the three replicates (Table 3). A total of 4297 proteins (70%) were identified in each replicate simultaneously. Only 1058 proteins uniquely present in one replicate among the confirmed proteins (Fig. 2a). Most proteins were mapped with at least two unique peptides in this study (Fig. 2b), which indicated the high reliability of our data. In the study, the presence of a lot of proteins with unknown function identified revealed the complex nature of the regulatory network involved in development.

**Table 3:** Summary of protein identification in ‘Ougan’ anther and ‘Wuzi Ougan’ anther

Database	No.	Total spectra	Spectra(PSM)	Peptides	Unique peptides	Protein groups
Cclementina	Replicate 1	390,756	68,360	27,181	25,108	5246
Cclementina	Replicate 2	386,419	69,962	26,298	24,243	5167
Cclementina	Replicate 3	389,202	67,492	26,323	24,238	5228
Cclementina	Total	1,166,377	205,814	37,967	35,036	6201

**Fig. 3:**
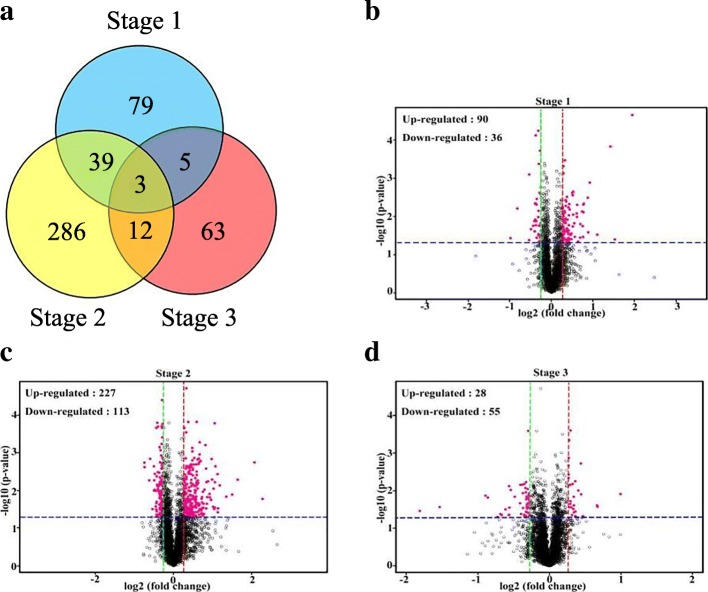
Analysis of the differentially expressed proteins (DEPs). **a** Venn Diagrams of DEPs in three stages. **b-d** Volcano plots of the DEPs in three stages. The green (down-regulated) and red (up-regulated) dotted vertical lines showed the threshold of fold change for 0.83 and 1.2, respectively, and the blue dotted horizontal lines indicated the threshold of p-value for 0.05. Dark red points refer to the proteins whose p-value was less than 0.05 and fold change of expression were more than 1.2 or less than 0.83 between MT and WT

Based on iTRAQ quantitative proteomics, we confirmed 6201 high-confidence proteins, 487 of which were DEPs (1.2-fold change, *p*-value < 0.05) in one or more stages (Additional file 1: Table S1). There were 126, 340, and 83 differentially expressed proteins in S1, S2, and S3 between MT and WT, respectively (Additional file 2: Table S2). Among the DEPs, only 3 proteins were shared by three stages, 56 proteins were shared by two stages, while only 79, 286, and 63 proteins had differential expression profiles in S1, S2, and S3, respectively (Fig. 3a). There were 90, 227, and 28 proteins up-regulated in MT in S1, S2, and S3, and 36, 113, and 55 proteins up-regulated in WT in S1, S2, and S3, respectively (Fig. 3b–d).

### COG and clusters of DEPs

**Fig. 4:**
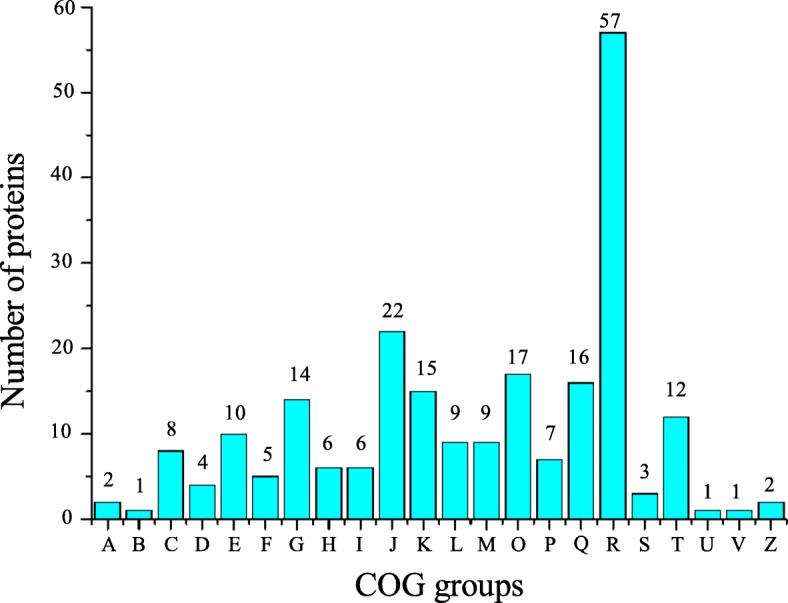
Functional classification of DEPs from anther proteome between ‘Ougan’ and ‘Wuzi Ougan’. A: RNA processing and modification, B: Chromatin structure and dynamics, C: Energy production and conversion, D: Cell cycle control, cell division, chromosome partitioning, E: Amino acid transport and metabolism, F: Nucleotide transport and metabolism, G: Carbohydrate transport and metabolism, H: Coenzyme transport and metabolism, I: Lipid transport and metabolism, J: Translation, ribosomal structure and biogenesis, K: Transcription, L:Replication、recombination and repair, M: Cell wall/membrane/envelope biogenesis, N: Cell motility, O: Posttranslational modification, protein turnover, chaperones, P: Inorganic ion transport and metabolism, Q: Secondary metabolites biosynthesis, transport and catabolism, R: General function prediction only, S: Function unknown, T: Signal transduction mechanisms, U: Intracellular trafficking, secretion, and vesicular transport, V: Defense mechanisms, W: Extracelluar structures, Y: Nuclear structure, Z: Cytoskeleton

To obtain a deeper understanding of the functions of the DEPs, a cluster of orthologous groups of proteins (COG) analysis was performed. As shown in Fig. 4, for more than half of the DEPs, which didn’t have COG or they belonged to the category with unknown function. A total of 181 DEPs were mainly classified into the categories of carbohydrate transport and metabolism; translation; ribosomal structure, and biogenesis; transcription; post-translational modification, protein turnover, and chaperones; secondary metabolite biosynthesis, transport, and catabolism; general function prediction only and signal transduction mechanisms, accounting for 14, 22, 15, 17, 16, 57, and 12 proteins, respectively. In addition, COG functions were largely enriched in the categories of translation, ribosomal structure, and biogenesis; transcription. Therefore, we consider that male sterility in MT may be influenced by genetic information processing, which might be related to abnormal meiosis. The analysis of these DEPs should improve our understanding of the mechanism behind male sterility in MT.

**Fig. 5:**
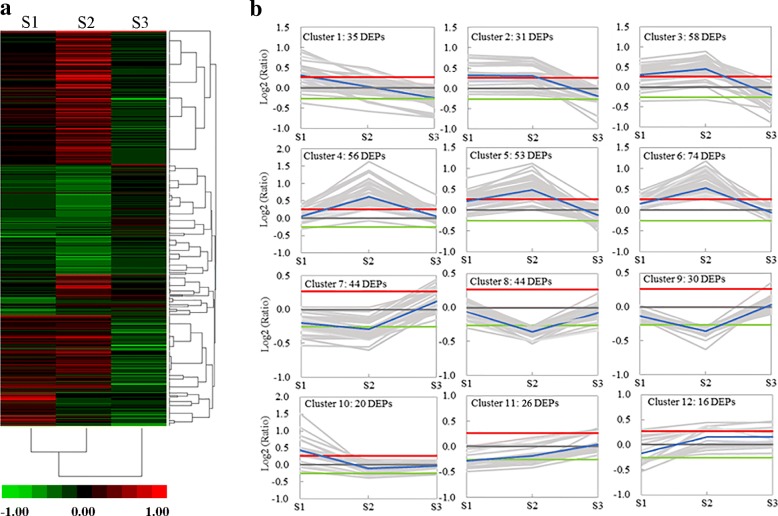
Expression profiles of DEPs in three development stages of anther between ‘Wuzi Ougan’ and ‘Ougan’. **a** Heat map for cluster analysis of the differentially expressed proteins by K-means method. Color indicates the fold change (red indicates up-regulated; green indicates down-regulated). **b** Mev cluster analysis of differentially expressed proteins from the protein expression profiles. The blue line is the mean of Log2(ratio) in each cluster, the red and green lines indicate cut-off of up or down protein regulated, respectively. Ratio indicated the ratio of average abundance between MT and WT at each stage. S1, S2, S3 are three developmental stages during microsporogenesis according to the length of floral bud: S1 (Stage 1) indicates the stage when the microsporogenesis initiated from sporogonium with bud length of 1.2 mm–1.6 mm, S2 (Stage 2) represents the stage when the microsporogenesis developed into the early microsporocyte with bud length of 2.0 mm–2.4 mm, and S3 (Stage 3) refers to the stage when the microsporogenesis entered into meiosis with bud length of 2.4 mm–2.8 mm

Protein expression during the stages of anther development was quantitatively analyzed in two strains. A total of 487 significantly differentially expressed proteins were identified and analyzed using the criteria of 1.2-fold change and a *p*-value < 0.05 (Fig. 5a). To obtain a deep understanding of the major trends of DEPs, we used a K-means method with a Euclidean distance metric using the software MultiExperiment Viewer. The DEPs were divided into 12 clusters (Fig. 5b). Among these DEP clusters, cluster 1 was particularly up-regulated at stage 1 and subsequently down-regulated. Clusters 2–6 showed the similar patterns, which were significantly up-regulated and reached their peak levels at stage 2. In clusters 2 and 3, DEPs were down-regulated at stage 3. A variety of DEPs in clusters 8–10 were significantly down-regulated in stages 1 and 2. Compared with cluster 10, cluster 12 showed the completely opposite trend.

### GO and KEGG analyses

Gene Ontology (GO), an international standardized protein functional classification system, is a significant tool to classify the functions of a lot of proteins. GO analysis has been widely applied to predict the functions of proteins in many organisms. The GO database is composed of three ontologies: molecular function (MF), cellular component (CC), and biological process (BP).

In this study, among the proteins within the category of biological process found to be differentially expressed in ‘metabolic process’ and ‘cellular metabolic process’, indicating that these proteins might intensively take part in assimilation and/or dissimilation involved in pollen development. In cellular components, ‘cell’ and ‘cell part’ were the predominant components of the category. Regarding the molecular function category, the main enriched subcategories were ‘catalytic activity’ and ‘binding’ in the key period of floral bud development (Additional file 3 Table S3).

**Fig. 6:**
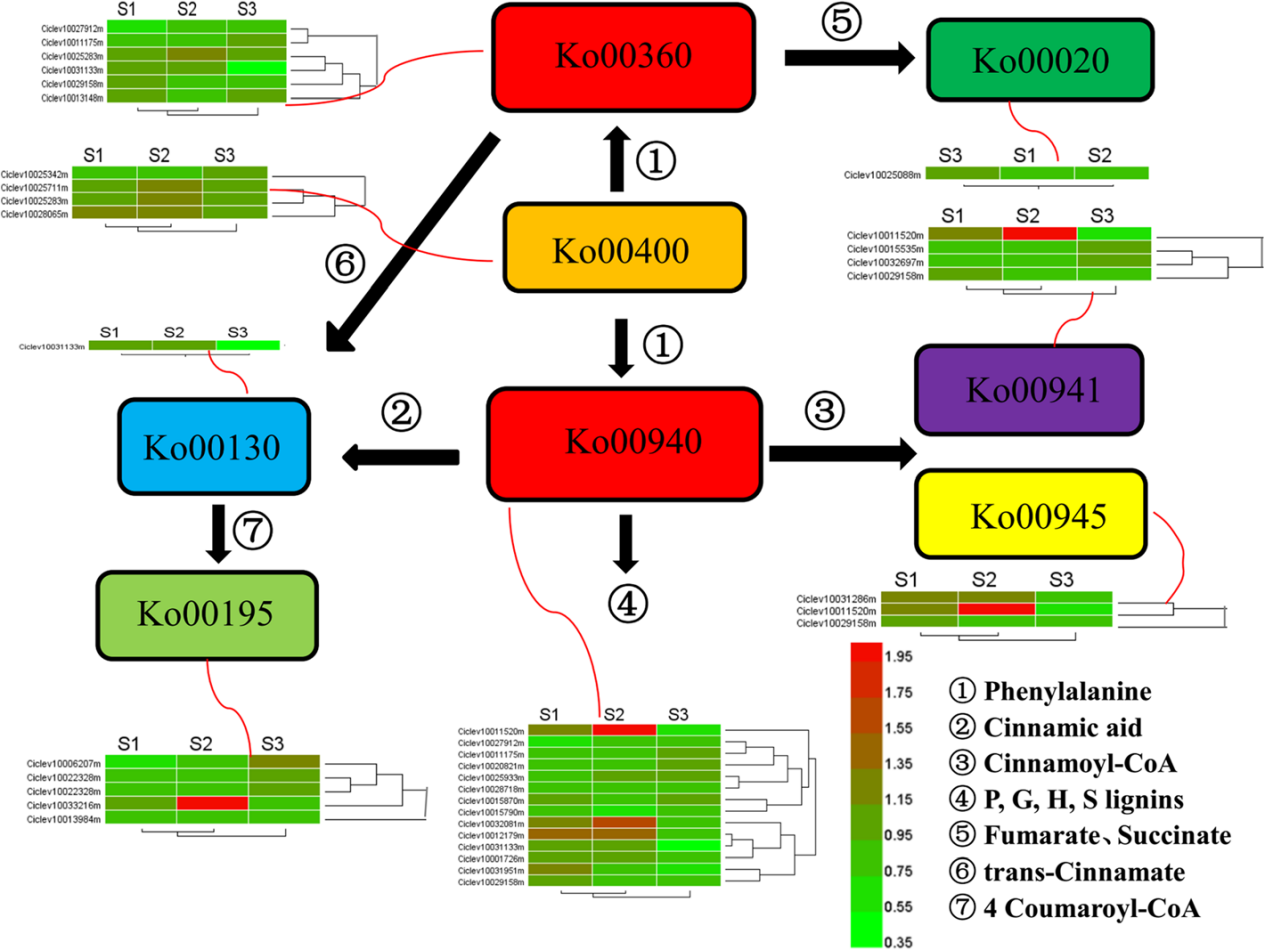
The protein abundance changes in the network of phenylpropanoid metabolism. S1, S2, S3 indicates the developmental stages of sporogonium, early microsporocyte and meiosis during microsporogenesis according to the length of floral bud, respectively. Enriched Pathways involved in phenylpropanoid metabolism were denoted as Ko group with color boxes: ko00020 (Citrate cycle), ko00130 (Ubiquinone and other terpenoid-quinone biosynthesis), ko00195 (Photosynthesis), ko00360 (Phenylalanine metabolism), ko00400 (Phenylalanine, tyrosine and tryptophan biosynthesis), ko00940 (Phenylpropanoid biosynthesis), ko00941 (Flavonoid biosynthesis), ko00945 (Stilbenoid, diarylheptanoid and gingerol biosynthesis). Abundance changes of hit DEPs in the pathways were showed with red (up-regulated) and green (down-regulated) heat map when compared between MT and WT in each stage. Numbers 1–7 refer to the product generated from each relevant pathway

KEGG, a major biological process database, contains seven categories: metabolism, genetic information processing, environmental information processing, cell process, biological system, human diseases, and drug development. To further reveal the metabolic pathways of MT involved in male sterility, an analysis of the pathways of DEPs was performed and predicted a total of 487 proteins in 175 metabolic pathways (Additional file 4 Table S4). There were 19 common pathways in three stages (Table 4). Among those, four were phenylalanine-related pathways, namely, phenylpropanoid biosynthesis (ko00940); phenylalanine metabolism (ko00360); stilbenoid, diarylheptanoid, and gingerol biosynthesis (ko00945); and flavonoid biosynthesis (ko00941). Another four pathways also related to phenylalanine pathways were associated with numerous DEPs: phenylalanine, tyrosine, and tryptophan biosynthesis (ko00400); ubiquinone and other terpenoid-quinone biosynthesis (ko00130); TCA cycle (ko00020); and oxidative phosphorylation (ko00190). These eight pathways related to this process are described in a metabolic network (Fig. 6). Phenylalanine was supplied to the network of the phenylpropanoid metabolism by pathway ko00400. Some phenylalanine enters ko00360, which enables the supply of fumarate and succinate to ko00020 and trans-cinnamate to ko00130. The rest of the phenylalanine enters ko00940, which supplies cinnamic acid and cinnamoyl-CoA to the downstream pathways ko00941, ko00945, and ko00130.

**Table 4:** The number of DEPs in different Pathways in three stages

KEGG pathway		Stage 1	Stage 2	Stage 3	Total
Map ID	Map Name				
ko00940	Phenylpropanoid biosynthesis	5	8	2	14
ko00360	Phenylalanine metabolism	2	3	2	6
ko00941	Flavonoid biosynthesis	2	2	1	4
ko00945	Stilbenoid, diarylheptanoid and gingerol biosynthesis	2	1	1	3
ko04075	Plant hormone signal transduction	1	4	1	4
ko03010	Ribosome	3	12	2	17
ko03040	Spliceosome	2	10	7	19
ko00520	Amino sugar and nucleotide sugar metabolism	3	5	1	8
ko03013	RNA transport	2	8	2	12
ko04141	Protein processing in endoplasmic reticulum	2	9	1	11
ko00500	Starch and sucrose metabolism	2	7	1	9
ko00230	Purine metabolism	2	5	1	7
ko04626	Plant-pathogen interaction	2	1	1	4
ko04612	Antigen processing and presentation	1	3	1	5
ko05166	HTLV-I infection	1	2	1	4
ko03015	mRNA surveillance pathway	1	3	1	5
ko00195	Photosynthesis	1	4	1	5
ko05200	Pathways in cancer	1	2	1	4
ko04111	Cell cycle - yeast	1	2	1	4

### Expression analysis of unigenes according to their cognate DEPs by qRT-PCR

**Fig. 7:**
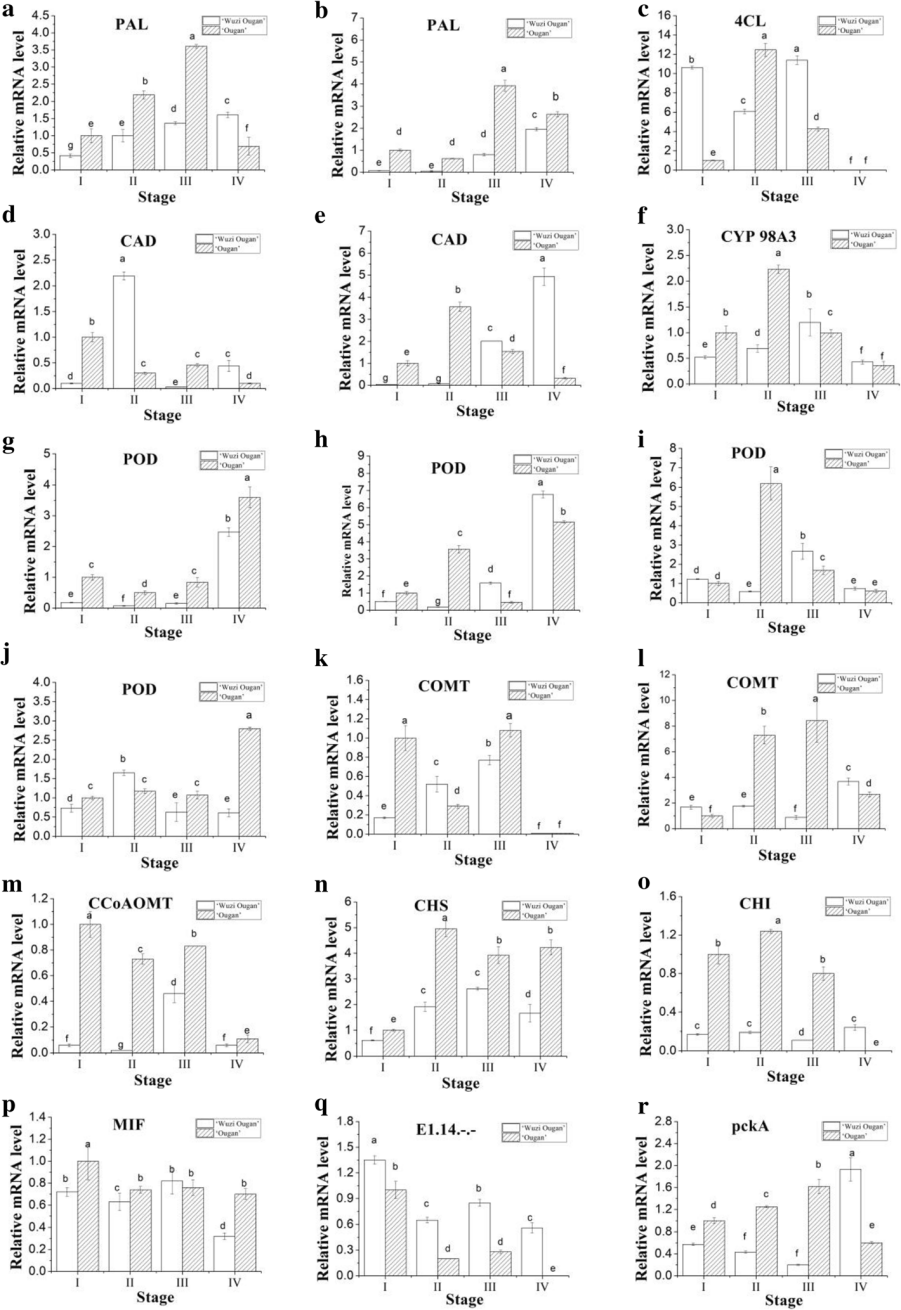
Relative abundance alterations of genes according to their DEPs involved in phenylpropanoid metabolism were compared by real-time quantitative PCR in anther of MT (white column) with WT (shadow column). I: anthers at the early microsporocyte stage. II: anthers at the stage of microsporocyte to meiosis. III: anthers at the tetrad stage. IV: anthers at the pollen maturation stage. **a**: Ciclev10027912m (PAL); **b**: Ciclev10011175m (PAL); **c**: Ciclev10031133m (4CL); **d**: Ciclev10028718m (CAD); **e**: Ciclev10025933m (CAD); **f**: Ciclev10011520m (CYP450 98A3); **g**: Ciclev10001726m (POD); **h**: Ciclev10015870m (POD); **i**: Ciclev10015790m (POD); **j**: Ciclev10032081m (POD); **k**: Ciclev10020821m (COMT); **l**: Ciclev10031951m (COMT); **m**: Ciclev10029158m (CCoAOMT); **n**: Ciclev10015535m (CHS); **o**: Ciclev10032697m (CHI); **p**: Ciclev10013148m (MIF); **q**: Ciclev10031286m (E1.14.-.-.); **r**: Ciclev10025088m (pckA). The data were analyzed by three independent repeats, and standard deviations were shown with error bars. Multiple comparisons were conducted between all samples in one gene and the small a-f indicated the significant difference (*p* < 0.05) when compared with each other

Eighteen unigenes, according to their cognate DEPs involved in phenylpropanoid metabolism identified by iTRAQ, were used for qRT-PCR analysis in terms of four developmental stages (I, II, III, IV) in MT and WT. Those genes include two *PAL* genes (phenylalanine ammonia-lyase), one *4CL* gene (4-coumarate-CoA ligase), two *CAD* genes (cinnamyl alcohol dehydrogenase), one *CYP98A3* gene (cytochrome 98A3), four *POD* genes (peroxidase), two *COMT* genes (caffeoyl-O-methyltransferase), one *CCoAOMT* gene (caffeoyl-CoA O-methyltransferase), one *CHS* gene (chalcone synthase), one *CHI* gene (chalcone isomerase), one *MIF* gene (migration inhibitory factor), and two other genes. The expression levels of these genes basically showed a similar trend to the pattern of their cognate proteins (Fig. 7).

### Integrative analysis of transcriptome and proteome

**Fig. 8:**
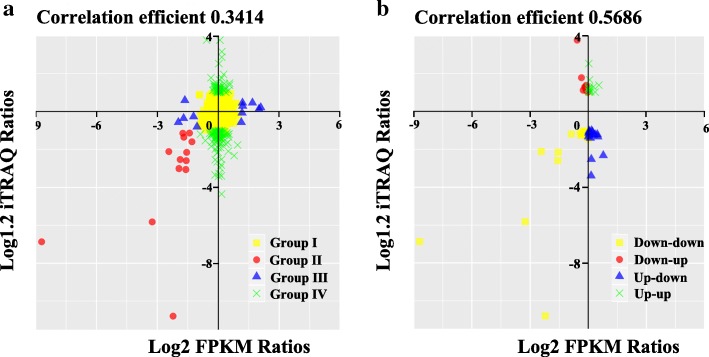
Correlation analysis between changes on protein levels (log1.2 iTRAQ ratios) in stage 3 and transcript levels (log2 FPKM ratios) for MT over WT. **a**: All proteins and all corresponding genes. **b**: DEPs and corresponding genes

An integrative analysis of transcriptome and proteome data was performed to identify the regulation of metabolism in microsporogenesis in MT and WT. The transcriptome data was obtained previously (https://www.ncbi.nlm.nih.gov/bioproject/?term=PRJNA430695) from anthers at microsporocyte stage. Therefore, the proteome data at S3 stage (microsporocyte to meiosis) were drew out to compare with the previous transcriptome. In this connection, a total of 3809 proteins were used to analyze synthetically, and 2809 of them hit their corresponding mRNAs in the transcriptome. Among these, there were 1585 (56.4%) protein–mRNA pairs that showed a consistent trend in both transcriptome and proteome (Additional file 5 Table S5). Total of 2809 protein-mRNA pairs were divided into four categories based on the mRNA level (log2 FPKM ratios) and protein level (log1.2 iTRAQ ratios) expression profiles. Group I consisted of 2632 proteins showing no change between transcriptome and proteome. There were 12 proteins and 152 proteins changed either in the transcriptome or the proteome in Group III and Group IV, respectively. There were 13 differentially expressed proteins that showed a consistent trend in transcription and proteome in Group II. In general, the expression tendencies of all genes and all proteins were positively correlated, in which the correlation coefficient was 0.3414 (Fig. 8a). Additionally, expression changes at transcript (log2 FPKM ratios) and protein (log1.2 iTRAQ ratios) levels were plotted for DEPs detected by proteome and their according genes, and the correlation coefficient was 0.5686 (Fig. 8b).

**Fig. 9:**
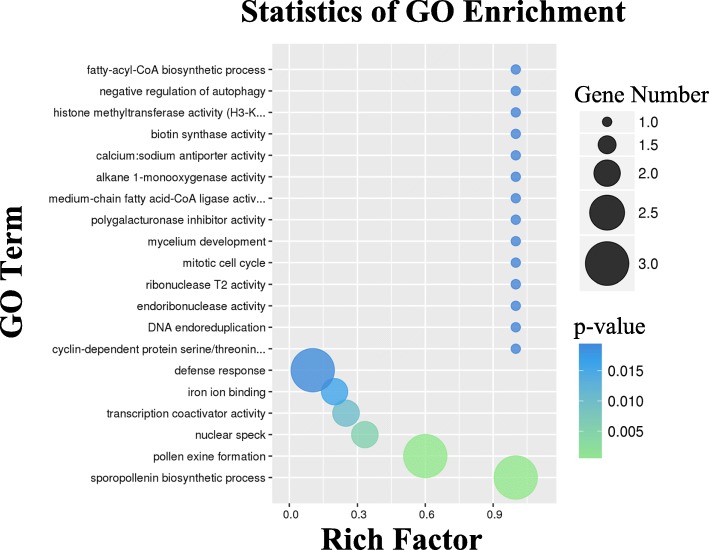
The statistics of GO enrichment for integrative analysis of all genes and differently expressed proteins. Numbers indicate amounts of proteins differently expressed matching cognate genes involved in relevant GO categories

**Fig. 10:**
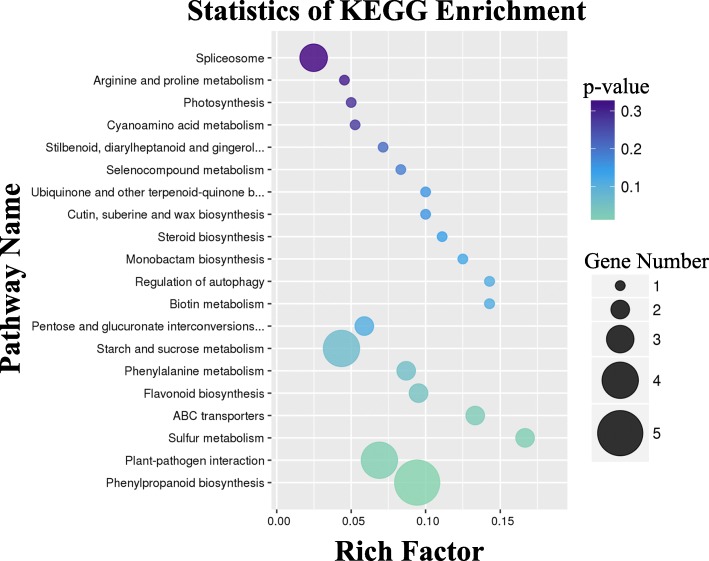
The statistics of KEGG enrichment for integrative analysis of all genes and differently expressed proteins. Numbers indicate amounts of proteins differently expressed matching cognate genes involved in relevant pathways

Correlative DEPs succeeded mapping to genes from RNA-Seq data were mainly involved in sporopollenin biosynthetic process, pollen exine formation, nuclear speck, and transcription coactivator activity (*p*-value< 0.01, Fig. 9). The enriched KEGG pathways included those related to metabolic pathways, such as phenylpropanoid biosynthesis (ko00940), plant-pathogen interaction (ko04626), sulfur metabolism (ko00920), ABC transporters (ko02010), flavonoid biosynthesis (ko00941), phenylalanine metabolism (ko00360), and starch and sucrose metabolism (ko00500) (p-value < 0.1, Fig. 10). Pathways related to phenylpropanoid metabolism were also classified into four categories (Table 5). Phenylpropanoid biosynthesis (ko00940) includes the most amount proteins and many DEPs in Group II and IV.

**Table 5:** Four groups proteins involving in the network of Phenylpropanoid metabolism

Pathway	ALL	I	II	III	IV
Ko00940	53	46	1	0	6
Ko00941	21	17	2	0	2
Ko00360	23	21	1	0	1
Ko00130	10	9	1	0	0
Ko00400	22	22	0	0	0
Ko00190	56	55	0	0	1
Ko00945	14	12	0	0	2
Ko00020	32	32	0	0	0

## Discussion

The development of flower organs is a highly coordinated and irreversible phenomenon that involves a series of physiological, biochemical, and organoleptic changes [28]. In the present study, proteome profiling was employed to investigate the differences between the mutant type and its wild type by iTRAQ technologies, where there were 487 DEPs showing variations in abundance during pollen development in the mutant. Using previous transcriptome data obtained by RNA-Seq, an integrative analysis was conducted to reveal the crosstalk between the transcriptome and the proteome in the regulation of pollen development, especially regarding male sterility in the mutant. Pathway enrichment was analyzed to reveal the importance of phenylpropanoid metabolism in this context, which has been widely reported to be involved in male sterility in plants [37, 38].

### Crosstalk between transcriptome and proteome responsible for male sterility

Integrative analysis of expressed genes and their cognate proteins improved our understanding of male sterility in the mutant. Four categories based on the mRNA level (log2 FPKM ratios) and protein level (log1.2 iTRAQ ratios) expression profiles were analyzed in detail. In general, there were 13 DEPs of which the expression was consistent with that of homologous DEGs in Group II (Fig. 8a), which suggests that those affected biological processes or pathways might be responsible for the male sterility in the mutant. The involved biological processes include pollen development (Ciclev10028834m), ATPase activity (Ciclev10010223m), pollen exine formation (Ciclev10010274m), hydrolase activity (Ciclev10016019m), 4-coumarate-CoA ligase (Ciclev10031133m), and lipid binding (Ciclev10006307m). The formation of pollen floral organ development. Among these DEPs and DEGs, *4CL* as a key enzyme in phenylpropanoid biosynthesis. qRT-PCR verification also showed that *4CL* is up-regulated in early microsporocytes (Fig. 7c). In rice, overexpression of the 4-coumarate-CoA ligase (*4CL*) related gene *OsAAE3* resulted in an increase in the content of H_2_O_2_ and led to programmed cell death (PCD) of the tapetum, which contributed to the suppression of floret development and decreased fertility rate of anther [52]. Male sterility is associated with premature or delayed PCD of the tapetum, which is the innermost layer of the anther and produces substantial nutrition for the development of microspore mother cell [53, 54]. Additionally, there were 12 genes assigned to Group III with expression changed at the transcript level and 152 genes changed at the peptide level (Group IV). Groups IV include many DEPs, such as peroxidase activity (Ciclev10001726m), lignin biosynthetic process (Ciclev10029158m) and cinnamyl-alcohol dehydrogenase activity (Ciclev10028718m). Lack of *CCoAOMT* (Ciclev10029158m) and *CAD* (Ciclev10028718m) impacts on lignification in the anther endothecium, which has been shown to be responsible for the failure of anther dehiscence and pollen release [41, 55]. The related genes were down-regulated in MT in the first two stages, as verified by qRT-PCR (Fig. 7d, m). Moreover, we observed the failure of anther dehiscence and pollen release in MT by stereoscope (Fig. 1c-d). *POD* can prevent the excessive accumulation of MDA and ROS enzymes by decomposing H_2_O_2_ into O_2_. Male sterility was reported to be due to dysfunction of the balance of *POD* content in floral organs [56]. *POD* is the last key enzyme in lignin synthesis, and down-regulated of Ciclev10001726m might ultimately influence lignin content in anther in ‘Wuzi Ougan’ before the tetrad stage in this research [57] (Fig. 7g).

This integrative analysis was focused on DEPs, and the genes associated with were according to the background of all genes obtained from transcriptome. However, the transcript levels indicated poorly their according proteins between MT and WT. Therefore, there were some possibilities for the inconsistent expression. It was possible that the materials used for sequencing were sampled from the same trees at different years. The expression levels between years changed to some extent. In addition to biological samplings, protein levels are regulated by posttranscriptional, translational, and post-translational mechanisms, and feedback loops exist between the processes of mRNA translation and protein degradation [58].

### Potential pathways related to male sterility in ‘Wuzi Ougan’

**Fig. 11:**
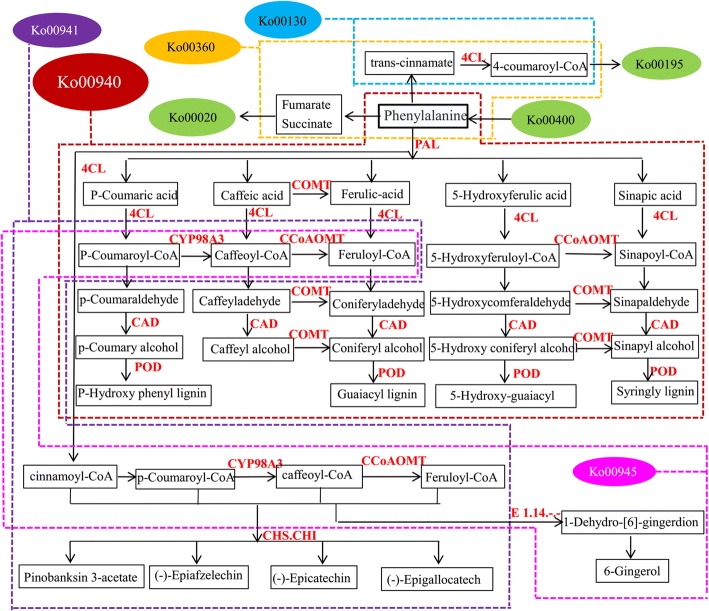
The phenylpropanoid metabolism in detail. The arrow tip indicates orientation of the substance transformation. The capital words in red indicates the enzymes catalyzed in the relevant pathway. The products linked with pathways of up- and down-stream were showed in black boxes. Color ellipses with ko numbers indicated the names of the pathways, and the products involved in each pathway were surrounded by the dotted line in colors according to their color index. The color index of dotted lines was as following: dark red, pink, purple, blue, yellow indicates pathway of ko00940 (Phenylpropanoid biosynthesis), ko00945 (Stilbenoid, diarylheptanoid and gingerol biosynthesis), ko00941 (Flavonoid biosynthesis), ko00130 (Ubiquinone and other terpenoid-quinone biosynthesis) and ko00360 (Phenylalanine metabolism), respectively. Green was used to indicate the ko00020 (Citrate cycle), ko00400 (Phenylalanine, tyrosine and tryptophan biosynthesis) and ko00190 (Oxidative phosphorylation), respectively

Genes and proteins revealed by the integrative analysis shown in Fig. 11 were particularly associated with phenylpropanoid biosynthesis (ko00940), and that these cooperated with other pathways as well as produced various secondary metabolites.

Phenylpropanoid biosynthesis, which plays center role in the network of phenylpropanoid metabolism, is an important pathway involved in standing upright, long-distance transport of water [59], leaf growth [60] and the development of floral organs [61]. Weaken activities of the enzymes associated with this pathway was proposed to male sterility [29–32]. In this study, the expression of *PALs* was down-regulated in MT before the tetrad stage (Fig. 7a, b). *4CL* as a specific enzyme in ko00940 plays an irreplaceable role in this metabolic process [62]. As revealed by qRT-PCR analysis, the expression pattern of *4CL* showed a sharp drop from microsporocyte to meiosis (Fig. 7c). Different CoA was difficult to produce due to the lack of *4CL* (Fig. 11). The deficiency of this enzyme delayed phenylalanine usage for lignin synthesis and flavonoid biosynthesis, as well as resulted in male sterility [63]. Coumaroyl-CoA is converted to p-coumaroyl-CoA, caffeoyl-CoA, and feruloyl-CoA via a series of reactions catalyzed by *4CL*, *CYP98A3*, and *CCoAOMT* (Fig. 11). Accordingly, down-regulated expression of *4CL*, *CYP98A3* and *CCoAOMT* (Fig. 7c, f, m) in MT during the microsporocyte to meiosis, as well as shared situation of p-coumaroyl-CoA, caffeoyl-CoA, and feruloyl-CoA in pathways (including ko00940, ko00941, and ko00360), were presumably to interrupt pollen development due to kinds of CoA deficiency. *COMT* and *CCoAOMT* are two anther-specific genes, *CCoAOMT* mainly plays a role in vascular tissues of young stamens, while *COMT* acts on the endothecium and the epidermal layer of stamens [55]. In fact, many genes involved in the network of phenylpropanoid metabolism efficiently expressed in anther or tapetum cells, such as *PAL* [34–36], *CHI* [40], *CHS* [38, 39], *COMT* and *CCoAOMT* [55]. Those most widely reported sterile genes were *CHS* homologous genes (*D5* [64], *YY2* [65], *LAP5/LAP6* [66]). Furthermore, the *CHS* mutant was reported to change colors of anthers, for example, from yellow to white, and led to dysfunctional male sterility [39]. It was also reported that overexpression of the *CHS* gene leads to male sterility along with intense pigmentation on the surface of the anther [67]. Therefore, it is considered that suitable gene expression is a genetic buffering mechanism to ensure floral organ function and appearance throughout development [22].

In addition, there were two pathways, oxidation-reduction and TCA cycle, frequently reported in male sterility plants and involved in the network of phenylpropanoid metabolism. Mitochondria are necessary organelles for cellular energy production because they participate in many metabolic pathways including the pentose phosphate pathway (ko00030), oxidative phosphorylation (ko00195), and the TCA cycle (ko00020) [28]. Via the ko00360 pathway, phenylalanine is converted to fumarate, succinate, or trans-cinnamate, which can ensure the functioning of ko00020 and ko00195. In this study, numerous DEPs were enriched in these three pathways, which were down-regulated at the early stage of pollen development (Table 6).For example, PckA (Ciclev10025088m), as a key gene in ko00020, were down-regulated in MT (Fig. 7r), which might influence the inversion of oxaloacetate to phosphoenolpyruvate [68].

**Table 6:** Identification of Phenylpropanoid metabolism proteins in the development anther

Map ID and Map name	Enzyme and Definition	Protein ID	KO	Stage 1	Stage 2	Stage 3
Phenylpropanoid biosynthesis(ko00940)	CYP98A3 [EC:1.14.13.36]	Ciclev10011520m	K09754	1.32	2.08	0.70
	PAL phenylalanine ammonia-lyase [EC:4.3.1.24]	Ciclev10027912m	K10775	0.71	0.75	0.89
		Ciclev10011175m	K10775	0.76	0.78	0.95
	CAD cinnamyl-alcohol dehydrogenase [EC:1.1.1.195]	Ciclev10025933m	K00083	0.78	1.01	1.05
		Ciclev10028718m	K00083	0.82	0.88	0.89
	POD peroxidase [EC:1.11.1.7]	Ciclev10032081m	K00430	1.19	1.55	0.89
		Ciclev10012179m	K00430	1.40	1.48	0.89
		Ciclev10001726m	K00430	1.01	1.02	0.80
		Ciclev10015870m	K00430	0.98	0.82	1.03
		Ciclev10015790m	K00430	0.81	0.71	0.81
	COMT caffeic acid 3-O-methyltransferase [EC:2.1.1.68]	Ciclev10020821m	K13066	0.85	0.82	1.04
		Ciclev10031951m	K13066	1.18	0.78	0.64
	CCoAOMT caffeoyl-CoA O-methyltransferase [EC:2.1.1.104]	Ciclev10029158m	K00588	0.98	0.89	0.81
	4CL 4-coumarate-CoA ligase [EC:6.2.1.12]	Ciclev10031133m	K01904	1.00	1.14	0.35
Phenylalanine metabolism(ko00360)	PAL phenylalanine ammonia-lyase [EC:4.3.1.24]	Ciclev10027912m	K10775	0.71	0.75	0.89
		Ciclev10011175m	K10775	0.76	0.78	0.95
	PAT,AAT bifunctional aspartate aminotransferase and glutamate/aspartate-prephenate aminotransferase [EC:2.6.1.1;2.6.1.78;2.6.1.79]	Ciclev10025283m	K15849	1.06	1.22	1.00
	MIF migration inhibitory factor [EC:5.3.2.1]	Ciclev10013148m	K07253	0.95	0.80	1.12
	CCoAOMT caffeoyl-CoA O-methyltransferase [EC:2.1.1.104]	Ciclev10029158m	K00588	0.98	0.89	0.81
	4CL 4-coumarate-CoA ligase [EC:6.2.1.12]	Ciclev10031133m	K01904	1.00	1.14	0.35
Flavonoid biosynthesis (ko00941)	CYP98A3 [EC:1.14.13.36]	Ciclev10011520m	K09754	1.32	2.08	0.70
	CHS chalcone synthase [EC:2.3.1.74]	Ciclev10015535m	K00660	0.77	0.75	0.99
	CHI chalcone isomerase [EC:5.5.1.6]	Ciclev10032697m	K01859	0.87	0.83	0.95
	CCoAOMT caffeoyl-CoA O-methyltransferase [EC:2.1.1.104]	Ciclev10029158m	K00588	0.98	0.89	0.81
Stilbenoid, diarylheptanoid and gingerol biosynthesis (ko00945)	E1.14.-.-	Ciclev10031286m	K00517	1.16	1.22	0.95
	CYP98A3 [EC:1.14.13.36]	Ciclev10011520m	K09754	1.32	2.08	0.70
	CCoAOMT caffeoyl-CoA O-methyltransferase [EC:2.1.1.104]	Ciclev10029158m	K00588	0.98	0.89	0.81
Ubiquinone and other terpenoid-quinone biosynthesis(ko00130)	4CL 4-coumarate-CoA ligase [EC:6.2.1.12]	Ciclev10031133m	K01904	1.00	1.14	0.35
Phenylalanine, tyrosine and tryptophan biosynthesis(ko00400)	aroF, aroG, aroH 3-deoxy-7-phosphoheptulonate synthase [EC:2.5.1.54]	Ciclev10025342m	K01626	0.78	0.87	1.00
	aroB 3-dehydroquinate synthase [EC:4.2.3.4]	Ciclev10025711m	K01735	0.78	0.87	1.00
	PAT, AAT bifunctional aspartate aminotransferase and glutamate/aspartate-prephenate aminotransferase [EC:2.6.1.1;2.6.1.78;2.6.1.79]	Ciclev10025283m	K15849	1.06	1.22	1.00
	trpE anthranilate synthase component I [EC:4.1.3.27]	Ciclev10028065m	K01657	1.16	1.22	0.95
Citrate cycle (TCA cycle)(ko00020)	pckA phosphoenolpyruvate carboxykinase (ATP) [EC:4.1.1.49]	Ciclev10025088m	K01610	0.80	0.87	0.96
Oxidative phosphorylation(ko00190)	NDUFA8 NADH dehydrogenase (ubiquinone) 1 alpha subcomplex subunit 8	Ciclev10006207m	K03952	0.74	0.79	1.24
	ATPeF1D, ATP5D, ATP16 F-type H + −transporting ATPase subunit delta	Ciclev10022328m	K02134	0.81	0.79	1.05
	NDUFS5 NADH dehydrogenase (ubiquinone) Fe-S protein 5	Ciclev10033216m	K03938	1.13	2.01	0.84
	ATPeF1D, ATP5D, ATP16 F-type H + −transporting ATPase subunit delta	Ciclev10022328m	K02134	0.81	0.79	1.05
	ATPF0A, atpB F-type H + −transporting ATPase subunit a	Ciclev10013984m	K02108	0.94	0.82	0.90

The abbreviations of enzyme are shown through overstriking. The underline expression satisfied with the standard of the differentially expression proteins

Three pathways (ko00940, ko00941, ko00360) were found to be enriched in the integrative analysis, which coordinately function in the regulatory network. Among them, the pathway of ko00940 and ko00360 produce substances and ATPs for microsporocytes (Fig. 11). Interestingly, another pathway, ko00400, involved in primary metabolism was not enriched (Table 4), and proposed an abundant of phenylalanine in MT. Therefore, the insufficiency or down-regulation of *4CL* from microsporocyte stage to meiosis stage might result in disorder of ko00940 and ko00360, and subsequently led to dysfunction of the network of phenylpropanoid metabolism. Such important alterations of 4CL expression might contribute to the male sterility in MT.

It is extremely complicated to unravel the mechanism behind male sterility, and KEGG enrichment analysis has become essential to survey this issue [69, 70]. In the present study, we considered that disordered phenylpropanoid metabolism along with the pathways up- or down-streamed are the focus point of male sterility in ‘Wuzi Ougan’.

## Conclusion

This paper has presented a comprehensive analysis of male sterility in ‘Wuzi Ougan’ and its wild type. Through integrative transcriptome and proteome analysis, DEGs and DEPs were identified to be particularly associated with phenylpropanoid biosynthesis, flavonoid biosynthesis, and phenylalanine metabolism. Genes were analyzed by qRT-PCR to present their according proteins related to the phenylpropanoid metabolism. This study provides a deeper understanding of the mechanism behind male sterility in citrus as well as the bud mutant.

## Additional files

Additional file 1: **Table S1.** All identification proteins in three stages. “N” indicates MT in three stages, and “Y” indicates WT in three stages. (XLSX 2862 kb)

Additional file 2: **Table S2.** Annotated DEPs and their expression ratio (N/Y). “N” and “Y” indicate MT and WT, respectively. (XLS 181 kb)

Additional file 3: **Table S3.** GO term of DEPs. (XLS 127 kb)

Additional file 4: **Table S4.** KEGG term of DEPs. (XLS 98 kb)

Additional file 5: **Table S5.** Integrated_Table_mRNA_protein_Anno. (XLSX 999 kb)

## Abbreviations

BP: Biological process
CC: Cellular component
CMS: Cytoplasmic male sterility
COG: Cluster of Orthologous Groups of proteins
DEGs: Differentially expressed genes
DEPs: Differentially expressed proteins
DSB: Double strand break
FPKM: Reads Per kb per Million reads
GO: Gene ontology
ICAT: Isotope coded affinity tag labeling
iTRAQ: Isobaric Tags for relative and absolute quantitation
KEGG: Kyoto encyclopedia of genes and genomes
MF: Molecular function
PCD: Programmed cell death
qRT-PCR: Real-time polymerase chain reaction
SEM: Scanning electron microscopy
TEM: Transmission electron microscopy
